# Antimicrobial stewardship prospective audit and feedback improves uptake of intravenous to oral antimicrobial switch in oncology inpatients: a cohort study

**DOI:** 10.1017/ash.2026.10880

**Published:** 2026-10-05

**Authors:** Nikhil R. Singh, Tim Spelman, Jacob Howell, Chulong Luo, Kanon Machray, Thao Tran, Safeera Y. Hussainy, Chiao X. Lim, Senthil Lingaratnam, Abby P. Douglas, Karin A. Thursky, Gabrielle M. Haeusler

**Affiliations:** 1 Pharmacy Department, Peter MacCallum Cancer Centre, Australia; 2 National Centre of Infections in Cancer, Melbourne, VIC, Australia; 3 Department of Infectious Diseases, Peter MacCallum Cancer Centre, Melbourne, VIC, Australia; 4 Sir Peter MacCallum Department of Oncology, University of Melbourne, Melbourne, VIC, Australia; 5 Centre for Health Services Research, Peter MacCallum Cancer Centre, Melbourne, VIC, Australia; 6 School of Medicine, Adelaide University, Adelaide, SA, Australia; 7 Department of Clinical Neuroscience, Karolinska Institute, Stockholm, Sweden; 8 Pharmacy, School of Health and Biomedical Sciences, Royal Melbourne Institute of Technology: RMIT University, Australia; 9 Department of General Practice, School of Public Health and Preventative Medicine, Monash University, Melbourne, VIC, Australia; 10 Department of Infectious Diseases and Immunology, Austin Health, Melbourne, VIC, Australia

## Abstract

**Objective:**

To evaluate the impact of antimicrobial stewardship (AMS) prospective audit and feedback on the timeliness of intravenous (IV) to oral (PO) antimicrobial switch among medical and surgical oncology inpatients.

**Design:**

Retrospective cohort study.

**Setting:**

Single center, public, quaternary cancer center.

**Patients:**

Medical and surgical cancer inpatients.

**Methods:**

We conducted a retrospective cohort study (March–August 2024) following implementation of a local IV to PO switch guideline. AMS prospective audit and feedback (AMS PAF) were rotated between medical and surgical oncology cohorts each month. Outcomes in patients receiving AMS PAF were compared to those managed by treating teams alone. The primary outcome was IV to PO switch within 24 hours of eligibility. Secondary outcomes included clinical end points, length of stay (LOS), antimicrobial consumption, and nursing administration time. A time-and-motion study quantified nursing time for IV and PO antimicrobial administration.

**Results:**

Among 204 hospital encounters and 554 antimicrobial prescriptions, AMS PAF was associated with higher rates of timely IV to PO switch (72.5% vs 40.8%), lower length of therapy and inpatient days of therapy/patient days, and reduced nursing administration time. There were no differences in LOS, 30-day readmission, ICU admission, mortality or 90-day *C. difficile* infection. IV to PO switch recommendations were highly accepted (73.3% (22/30)).

**Conclusions:**

In medical and surgical oncology inpatients, AMS PAF improved adherence to the local IV to PO switch guideline, reduced antimicrobial use and nursing administration time without an adverse impact on key clinical outcomes. AMS PAF offers a safe, effective, and resource-efficient strategy in cancer care.

## Introduction

Infections are a significant cause of morbidity and mortality in patients with cancer.^1^ Although it is well understood that systemic chemotherapy increases the risk of infection through immune impairment and gastrointestinal mucosal disruption, novel targeted treatments (immune, oral targeted and cellular therapies)^2^ and other cancer treatments (radiation therapy and surgery) are also associated with significant infection-related complications.^3–5^ As a result, patients with cancer have more frequent interactions with healthcare and consume more antimicrobials relative to those without cancer.^6^ These factors increase the risk of colonization and infection with multidrug-resistant organisms (MDROs)^7^ which are becoming increasingly prevalent in Australia.^8^ Infections with MDROs compromise the efficacy of empiric antibiotics leading to adverse outcomes including treatment failure, prolonged hospitalizations, cancer treatment delays and mortality.^7^ These challenges highlight the need for antimicrobial stewardship (AMS) programs tailored to the complex oncology setting.

Prospective audit and feedback (PAF) is a core AMS activity^9^ that is associated with improved prescribing appropriateness.^10^ A growing focus of AMS PAF is facilitating early intravenous to oral antimicrobial switch (IV to PO switch) as emerging data support its safety and efficacy.^11^ Reported benefits of AMS prompted IV to PO switch include reduced length of stay (LOS), antibiotic consumption, hospital readmission and *C. difficile* infection.^12^ Further, a time-and-motion study has suggested that IV to PO switch reduces nursing administration time.^13^ However, data regarding the feasibility and safety of early IV to PO switch in patients with cancer, particularly in the Australian setting, are limited.^14^

The primary objective of this study was to evaluate the impact of AMS PAF on the timeliness of IV to PO switch among medical and surgical oncology inpatients compared to treating team-directed care. Secondary objectives were to evaluate antimicrobial consumption, LOS, clinical outcomes (unplanned readmission, ICU admission and mortality), and nursing time associated with antimicrobial administration. We hypothesized that AMS PAF would improve timeliness of IV to PO switch, reduce antimicrobial consumption and LOS without worsening clinical outcomes, after adjusting for key clinical confounders.

## Methods

### Study setting and description of routine AMS team practice

The Peter MacCallum Cancer Centre (Melbourne, Australia) is a quaternary 100-bed cancer center providing inpatient and outpatient hematology, medical oncology, surgical oncology and radiotherapy services.

The infectious diseases (ID) and AMS service are embedded throughout the hospital. The ID service provides a consult service at the request of treating teams and attend weekly interdisciplinary hematology ward rounds. The AMS team is responsible for local guidelines, audits and feedback, antimicrobial formulary restrictions and monitoring of antimicrobial consumption. The AMS team conduct twice weekly, pharmacist-led ward rounds providing antimicrobial PAF to the medical and surgical oncology teams. Ward rounds are also attended by a specialist nurse/nurse practitioner and a rotating ID physician. Patients who are receiving restricted IV antimicrobials (eg, piperacillin-tazobactam, meropenem), longer courses of IV therapy, have multiple comorbidities or are commenced on the institutional sepsis pathway are prioritized for AMS PAF. Following review, recommendations are documented in the electronic medical record (EMR). PAF recommendations are communicated to treating teams via an EMR secure chat function.

In October 2023, the AMS team implemented a hospital wide IV to PO switch guideline. Interdisciplinary education and promotion was conducted during this period. Following guideline implementation, the AMS team documented patient eligibility for IV to PO switch (Appendix 1) in the EMR following PAF.

Between 19/03/2024 and 23/08/2024, AMS ward rounds were rotated every four weeks between two inpatient streams, medical and surgical oncology. The intervention cohort comprised of inpatient admissions where AMS PAF occurred, and the comparator cohort comprised of inpatient admissions to the non-reviewed oncology team during the same rotational period and were managed by treating teams without AMS PAF (treatment team-directed care). For treatment team-directed care encounters, “potential review” was defined as the corresponding date on which AMS PAF would have occurred had that oncology stream been allocated to AMS PAF.

To assess impact of AMS PAF, a retrospective chart review was conducted to compare the patient cohorts who received AMS PAF to those who did not (treating team-directed care). Data was collected using an online database (REDCap®, Version 15.5.38, Vanderbilt University, Nashville, TN). Patients were included if they were prescribed an IV antimicrobial at the time of first AMS PAF or potential review. Patients were excluded if, at the time of first AMS PAF or potential review, the patient was being consulted by ID or if antimicrobials prescribed were for prophylaxis or for a non-infective indication.

### Data

Baseline characteristics including age, gender, admitting specialty and Charlson Comorbidity Index were collected. Antimicrobial prescription data including the antimicrobial name, route, dose/frequency, start and end dates and indication (categorized by the study team into respiratory, intra-abdominal, urinary/genitourinary, fever or suspected infection without defined clinical syndrome and others) were collected. For antimicrobial courses given for an indefinite duration (eg, suppressive therapy), the date of IV to PO switch was assigned as the end date.

For the AMS PAF encounters, the types of recommendations made for each prescription were recorded (cease antimicrobial, IV to PO switch, seek expert advice, change spectrum, change dose, commence antimicrobial prophylaxis or duration advice) as well as acceptance of recommendations (within 24 h, 24–72 h or not accepted).

For treating team-directed care encounters, the date of potential AMS review(s) was collected (ie, the dates a patient was on an IV antimicrobial where an AMS round occurred in the corresponding group). The IV to PO switch criteria was assessed retrospectively by the study team AMS pharmacist to determine if the patient was eligible for switch on the days of a corresponding AMS round.

### Outcomes

The primary outcome was the proportion of eligible IV antimicrobial prescriptions switched to oral therapy within 24 hours of meeting switch criteria (assessed at the time of first AMS review or potential review), excluding prescriptions where antibiotic therapy were ceased entirely.

Secondary outcomes included LOS, antimicrobial consumption, 90-day *C. difficile* infection and 30-day all-cause mortality, ICU admission, unplanned re-admission and antibiotic recommencement. The 30- and 90-day periods were measured from the date of the first AMS PAF or for those in the treatment team-directed care cohort, the first potential review.

Antimicrobial consumption was measured using length of therapy (LOT), days of therapy (DOT) and inpatient DOT/patient days. LOT was defined as the total number of calendar days during which a patient received any antimicrobial therapy regardless of the number of agents or their frequency. DOT was defined as the total number of calendar days on which a patient received at least one dose of an antimicrobial agent (with each antimicrobial counted separately), regardless of the number of doses administered per day. LOT and DOT included the number of days antibiotics were prescribed during the entire hospital admission and the intended duration following hospital discharge. Inpatient DOT/patient days was defined as the DOT accrued during inpatient admission only, divided by the number of days admitted to mitigate the impact of differences in LOS between cohorts.

### Time-and-motion study

A time-and-motion study was performed alongside the retrospective review; the aim was to quantify the nursing time taken to prepare and administer IV and PO antimicrobials. Nurses on the medical and surgical oncology wards were selected using convenience sampling and provided verbal consent to be observed from 8am to 11am from Wednesday to Friday over a four-week period (14/08/2024–06/09/2024).

Single observers measured the time taken to administer IV and PO medicines. This included the time taken from the point the nurse entered the medication room to find the antimicrobial to the time point where the medicine was marked as given in the clinical record. For IV antimicrobial infusions, the time the infusion ran was not included.

### Statistical analyses

Categorical variables were summarized using frequency and percentage. Continuous variables were summarized using mean, standard deviation (SD) and range; and/or median, interquartile range (IQR) and range as appropriate. Time-to-event outcomes were summarized and visualized using Kaplan-Meier survivor functions and survival and failure curves.

The primary outcome was analyzed using Cox proportional hazards regression. Effect sizes were expressed as hazard ratios (HRs) with 95% confidence intervals (CIs). Hazard proportionality was assessed using scaled Schoenfeld residuals. Univariate and multivariate models were fitted, with multivariate analyses adjusting for age, sex, specialty type (medical vs surgical oncology), cancer type (gastrointestinal, head and neck, lung, sarcoma and other) and comorbidities (defined by Charlson Comorbidity Index variables). Variable selection was based on clinical relevance and univariate model performance. Models were assessed for collinearity and interactions.

Secondary outcomes were analyzed using regression models appropriate to the outcome type. Antimicrobial consumption (LOT, DOT and inpatient DOT/patient days) were analyzed using negative binomial regression with follow-up time included as an offset where appropriate. Binary clinical outcomes were analyzed using logistic regression. Effect sizes were expressed as risk ratios (RR) with associated 95% CIs. The same covariates previously described were included in the analysis as adjusting confounder covariates.

A stabilized inverse probability of treatment weight (IPTW) analysis was performed as a sensitivity analysis to further account for baseline differences between the AMS PAF and treatment team-directed care cohorts. Propensity scores were estimated using a multivariate logistic regression model including the covariates listed previously. Stabilized inverse probability weights were calculated to estimate the average treatment effect and covariate balance before and after weighting was assessed using standardized mean differences (SMDs), with an absolute SMD < 0.10 considered indicative of adequate balance. IPTW analyses were considered sensitivity analyses, with results compared with the primary multivariate-adjusted analyses to assess the robustness of the findings.

Estimated cumulative nursing administration time and mean nursing administration time per inpatient encounter were derived by applying the observed mean IV and PO antimicrobial administration times from the time-and-motion study to the estimated numbers of IV and PO antimicrobial administrations in each cohort. Uncertainty around these estimates was quantified using non-parametric bootstrapping. IV (n = 20) and PO (n = 31) administration times were resampled independently with replacement over 100,000 iterations. For each bootstrap sample, mean IV and PO administration times were recalculated and applied to the observed administration counts to estimate cumulative nursing time and mean nursing time per encounter. Point estimates are presented with percentile-based 95% CIs.

For all analyses, *P* < .05 was considered significant. All analyses were undertaken in Stata version 18 (StataCorp. 2023. *Stata Statistical Software: Release 18.* College Station, TX: StataCorp LLC.).

Ethics approval was granted by the Peter MacCallum Cancer Centre Human Research Ethics Committee (HREC/108201/PMCC).

## Results

A total of 204 hospital encounters and 554 antimicrobial prescriptions were included from 19/03/2024 to 23/08/2024. Baseline patient characteristics of the cohorts are shown in Table 1.

**Table 1.** Patient characteristics in the treatment team-directed care and AMS prospective audit and feedback cohorts Table 1 long description.

	Treatment team-directed care	AMS PAF	SMD
Hospital encounters (n)	112	92	–
Patients (n)	108	87	–
Antimicrobial prescriptions (n)	330	224	–
Age (years) (median [IQR])	64.5 (53–72.5)	62.5 (47–72)	0.172
Male n(%)	68 (60.7)	49 (53.3)	0.136
Female n(%)	44 (39.3)	43 (46.7)	
Metastatic cancer n(%)	63 (56.3)	63 (68.5)	0.234
Charlson comorbidity index (median [IQR])	7 (5–9)	7 (5–9)	0.013
Medical oncology n(%)	56 (50.0)	59 (64.1)	0.288
Lung	17 (15.1)	10 (10.9)	0.136
Sarcoma	5 (4.5)	11 (12.0)	0.363
Head and Neck	8 (7.1)	7 (7.6)	0.028
Other	26 (23.2)	31 (33.7)	0.135
Surgical n(%)	56 (50.0)	33 (35.9)	0.288
Colorectal	30 (26.8)	18 (19.6)	0.170
Upper gastrointestinal	9 (8.0)	4 (4.4)	0.145
Skin	4 (3.6)	1 (1.1)	0.174
Sarcoma	2 (1.8)	3 (3.3)	0.090
Other	11 (9.8)	7 (7.6)	0.078
Antimicrobial at time of first review *n*(%)			
Meropenem	1 (0.9)	4 (4.3)	0.214
Piperacillin-tazobactam	59 (52.7)	46 (50.0)	0.054
Cefepime	5 (4.5)	4 (4.3)	0.009
Ceftriaxone	28 (25.0)	23 (25.0)	0.000
Amoxicillin-clavulanate (IV)	11 (9.8)	2 (2.2)	0.323
Other	8 (7.1)	13 (14.1)	0.220
Antimicrobial indication at time of first review *n*(%)			
Respiratory tract	104 (31.5)	72 (32.1)	0.013
Intra-abdominal	109 (33.0)	50 (22.3)	0.241
Urinary/genitourinary	16 (4.8)	28 (12.5)	0.273
Fever or suspected infection (clinical syndrome not identified)	60 (18.2)	40 (17.9)	0.008
Other	41 (12.4)	34 (15.2)	0.081

AMS PAF, antimicrobial stewardship prospective audit and feedback.

There was no difference in the median days [IQR] to first AMS PAF (1 [1–3] d) or potential review (1 [1–2] d) between cohorts (SMD 0.22, 95% CI −0.05, 0.50).

### AMS team recommendations

A total of 125 antimicrobial prescriptions were reviewed (including the first AMS PAF per encounter only) resulting in 168 recommendations, with multiple recommendations permitted per prescription. Overall, 57.8% (97/168) of recommendations were accepted within 24 hours and 69.1% (116/168) within 72 hours.

**Figure 1.**
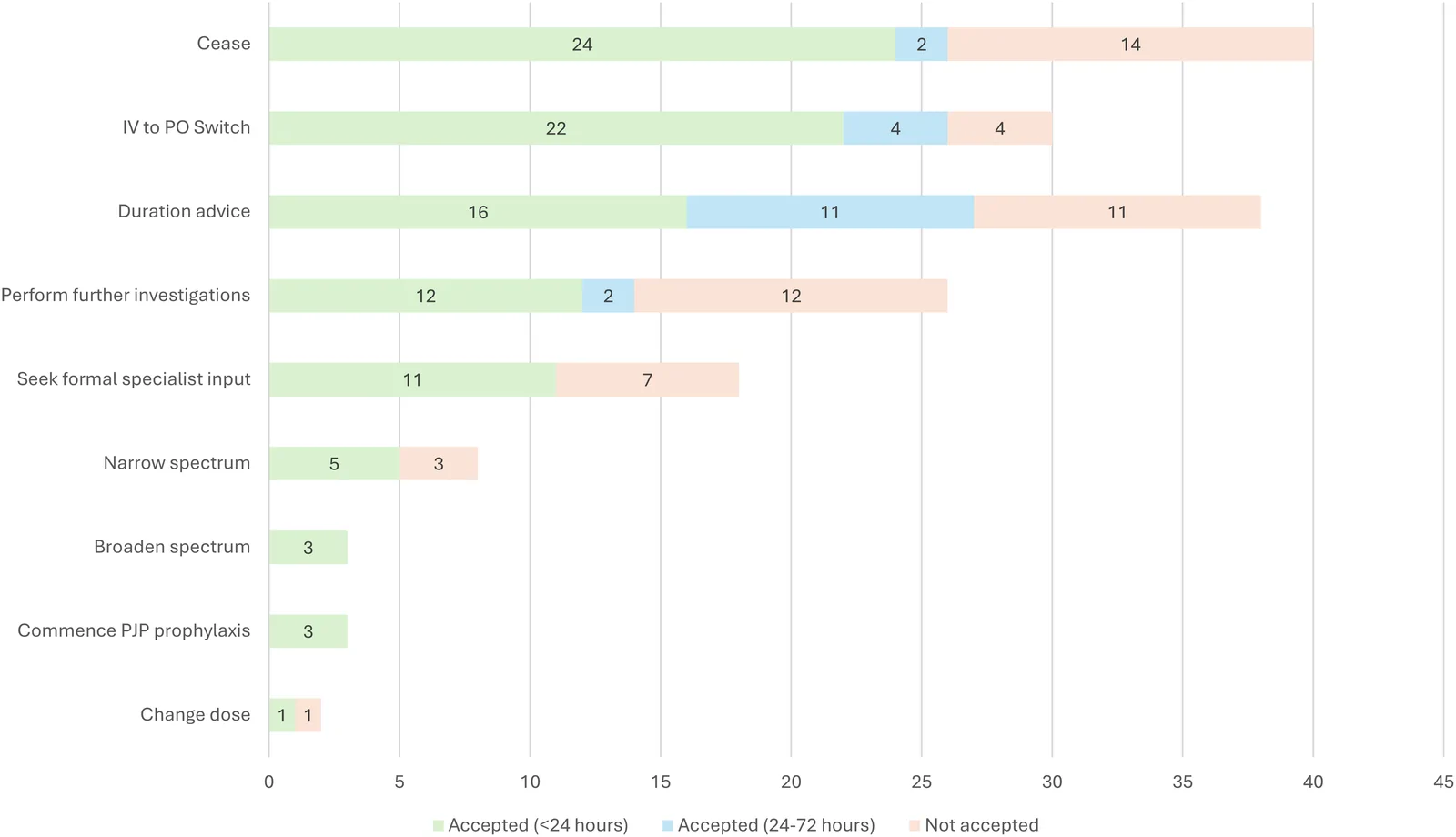
Antimicrobial stewardship team prospective audit and feedback recommendations (first review only, 168 recommendations in 92 encounters).

Recommendation types and acceptance are shown in Figure 1. Duration advice was provided in 83.3% (25/30) of IV to PO switch recommendations.

### Eligibility for IV to PO switch

At the time of the first AMS PAF or potential review, 44.2% (61/138) of prescriptions were eligible for IV to PO switch in the AMS PAF cohort and 38.9% (58/149) in the treatment team-directed care cohort.

In the AMS PAF cohort, 35% (21/60) of IV antimicrobial prescriptions eligible for switch had antimicrobial therapy ceased entirely within 24 hours compared to 15.5% (9/58) in the treatment team-directed care cohort.

### Primary outcome

**Figure 2.**
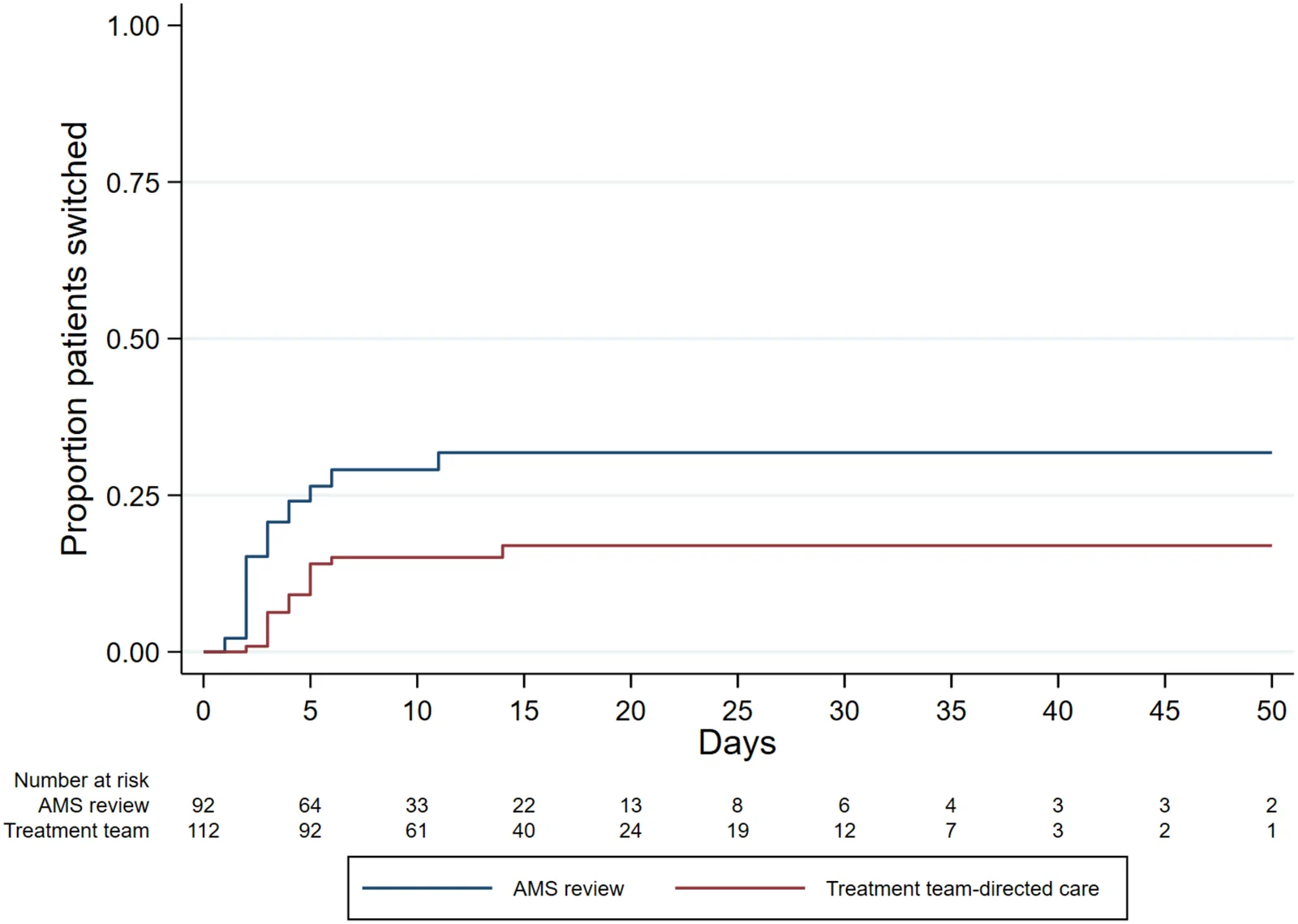
Proportion of patients switched following first AMS PAF or potential review.

Among eligible IV antimicrobial prescriptions that were not ceased, IV to PO switch within 24 hours of meeting eligibility criteria occurred more frequently in the AMS PAF cohort than in the treatment team-directed care cohort (72.5% [29/40] vs 40.8% [20/49]). Consistent with this, the time to IV to PO switch was significantly shorter in the AMS PAF cohort (adjusted HR 2.05, 95% CI 1.08–3.86; Figure 2), after adjustment for specialty type (medical vs surgical oncology), cancer type (sarcoma vs other) and chronic obstructive pulmonary disease (COPD).

### Secondary outcomes

#### Length of stay

There was no statistically significant difference in the median [IQR] LOS in the AMS PAF cohort (9.7 [5–17.1] d) compared to treating team-directed care cohort (12.8 [5.3–21.9] d) (*P =* .111).

#### Antimicrobial consumption

The most common IV antimicrobials prescribed were piperacillin-tazobactam (n = 151) and ceftriaxone (n = 72). Amoxicillin-clavulanate was more commonly prescribed PO (n = 122) than IV (n = 22).

Antimicrobial consumption in the treatment team-directed care and AMS PAF cohorts are shown in Table 2. Significantly lower IV antimicrobial LOT and inpatient DOT/patient days were observed in the AMS PAF cohort.

**Table 2.** Antimicrobial consumption (length of therapy (LOT), days of therapy (DOT) and inpatient DOT/patient days) adjusted for significant variables identified from specialty type, cancer diagnosis and Charlson comorbidity index Table 2 long description.

	Treatment team-directed care (n = 112 encounters)	AMS PAF (n = 92 encounters)	Adjusted β-coefficient (95% CI)	*p*-value
	Median [IQR]			
LOT (IV + PO)	11 [8–18]	8 [5.5–13.5]	−3.56 (−6.86, -0.27)	***.034***
PO	6 [3–9]	5 [0–7.5]	−1.9 (−4.37, 0.57)	*.131*
IV	6.5 [4–10]	5 [3–7]	−2.24 (−3.97, −0.52)	***.011***
DOT (IV + PO)	15 [10–22]	10.5 [6–17.5]	−4.38 (−8.89, 0.12)	*.057*
PO	6 [3–10]	5 [0–8]	−1.84 (−5.07, 1.38)	*.261*
IV	8 [5–12]	5 [3–8]	−2.37 (−5.09, 0.35)	*.087*
Inpatient DOT/patient days (IV + PO)	1.1 [0.7–1.4]	0.82 [0.6–1.37]	−0.19 (−0.35, −0.03)	***.017***
PO	.36 [0–.5]	.2 [0–.5]	0.01 (−0.09, 0.12)	*.840*
IV	.8 [.5–1.1]	.6 [.3–1]	−0.21 (−0.34, −0.08)	***.002***

LOT, length of therapy; DOT, days of therapy; AMS PAF, antimicrobial stewardship prospective audit and feedback. The bold values indicate statistically significant differences.

**Figure 3.**
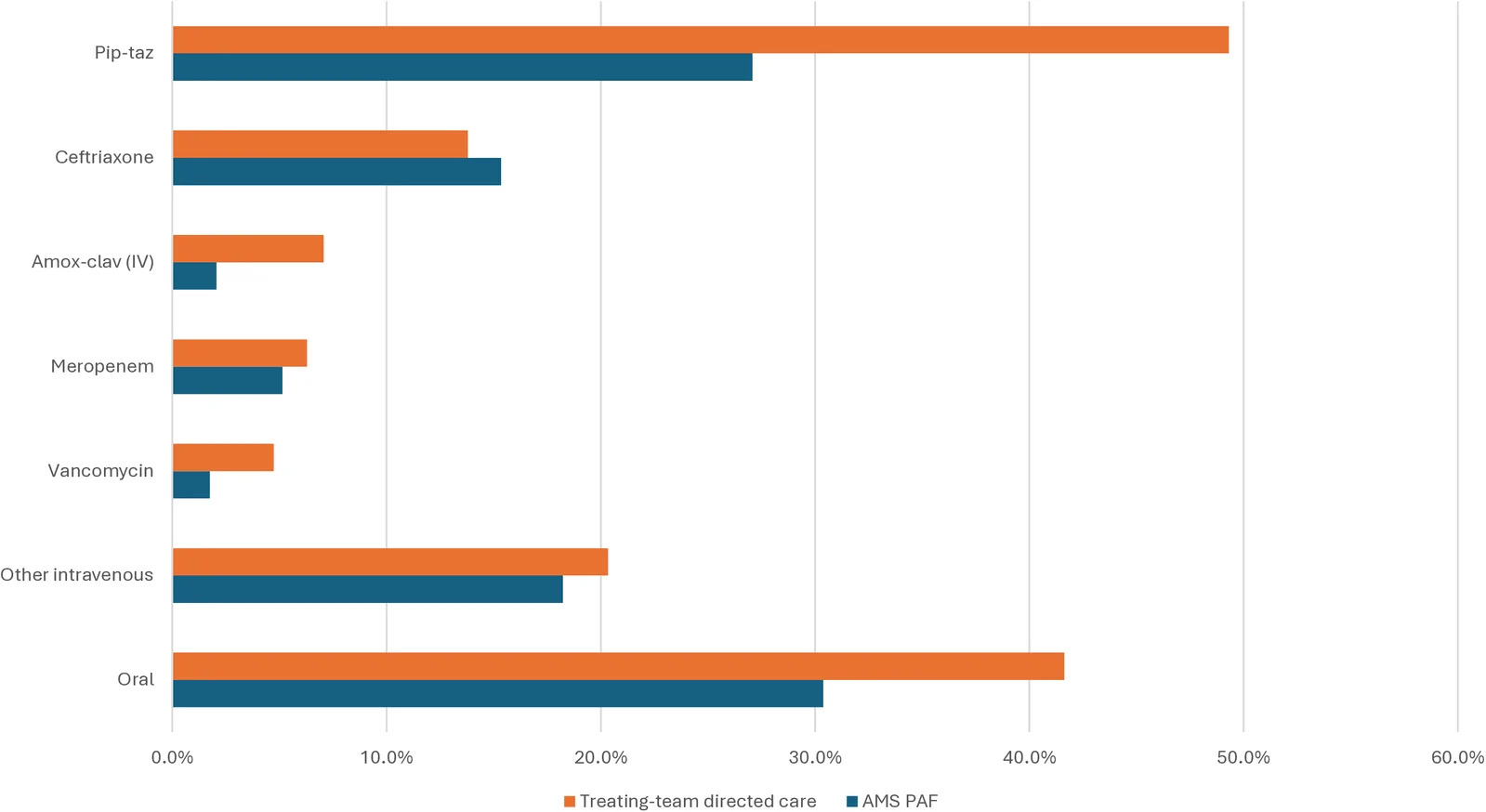
Antimicrobial use as a proportion of inpatient DOT/Patient days.

Abbreviations: Pip-taz: Piperacillin-tazobactam; Amox-clav: Amoxicillin-clavulanate; AMS PAF: Antimicrobial stewardship prospective audit and feedback.

Antimicrobial use as a proportion of inpatient DOT/patient days are shown in Figure 3. Lower consumption of anti-Pseudomonas beta-lactam antibiotics (eg, piperacillin-tazobactam and meropenem) were observed in the AMS PAF cohort.

#### Outcomes

Following multivariate analysis, there were no differences observed in 30-day mortality (adjusted for specialty type (surgical vs medical oncology)), unplanned hospital readmission (adjusted for age) and re-commencement of antibiotics (adjusted for age) (Table 3). No difference was observed in ICU admission following univariate analysis (insufficient event numbers to perform multivariate analysis).

**Table 3.** Clinical outcomes adjusted for significant variables identified from specialty type, cancer diagnosis and Charlson Comorbidity Index Table 3 long description.

	Treatment team-directed care	AMS PAF	Unadjusted OR (95% CI)	Adjusted OR (95% CI)	*p*-value
	**n/N (%)**		**Reference group: treatment team-directed care**		
Mortality (30-day)	16/108 (14.8)	16/87 (18.4)	1.36 (0.64, 2.87)	1.08 (0.49, 2.38)	.842
ICU admission (30-day)	5/112 (4.5)	3/92 (3.3)	0.72 (0.17, 3.11)	Not reported (insufficient numbers)	.662
Unplanned readmission (30-day)	13/104 (12.5)	20/88 (22.7)	2.06 (0.96, 4.43)	1.93 (.89, 4.20)	.072
Antibiotic re-commencement (30-day)	18/100 (18.0)	27/88 (30.7)	1.89 (0.96, 3.70)	1.80 (0.90, 3.58)	.095
*C. difficile* infection (90-day)	1/108 (0.9)	0/87 (0)	Not reported		

OR, Odds ratio, ICU, intensive care unit, AMS PAF, antimicrobial stewardship prospective audit and feedback.

#### Sensitivity analysis

Propensity weighting successfully balanced the treatment groups and after IPTW, all measured baseline covariates were balanced. The overall findings were consistent with the primary multivariate analyses. A higher proportion of AMS PAF cohort prescriptions that were eligible for IV to PO switch at the time of first AMS review or potential review were switched within 24 hours (HR 1.87 (1.02, 3.01) (*P = .*031). Total (intravenous and oral) and intravenous antimicrobial usage as measured by LOT, DOT and inpatient DOT/patients days were significantly shorter in the AMS PAF cohort (Supplementary Table 1). No differences in clinical outcomes were observed (Supplementary Table 2).

#### Time-and-motion study

The median [IQR] administration time for PO antimicrobials (n = 31) was 1 [0.5–4] minute compared to 10.2 [3–17.4] minutes for IV antimicrobials (n = 20)(*P < .001*). There were an estimated 3,446 IV and 990 PO doses administered in the treatment team-directed care cohort compared to 1,808 IV and 602 PO doses in the AMS PAF cohort.

Cumulative nursing time spent administering antimicrobials was 602.1 (95% CI 500.4, 694) in the treatment team-directed care cohort and 315.4 (95% CI 264.1, 365.7) hours in the AMS PAF cohort, representing an estimated saving of 286.7 hours (95% CI 236.3, 328.2). The mean nursing administration time per inpatient encounter was 5.4 hours (95% CI 4.47, 6.20) and 3.4 hours (95% CI 2.87, 3.98), respectively.

## Discussion

In this retrospective cohort study of oncology inpatients, AMS PAF was associated with substantially higher uptake of timely IV to PO antimicrobial switch compared with treating team-directed care. The intervention was also associated with lower intravenous antimicrobial exposure and reduced estimated nursing administration time, without statistically significant differences in LOS, readmission, mortality, ICU admission, or antimicrobial recommencement.

Randomized controlled trials have consistently demonstrated that PO antibiotics are safer and as effective as IV antibiotics.^11^ Consequently, there is a growing interest in implementing early IV to PO switch strategies. We observed higher rates of early IV to PO switch in patients receiving AMS PAF. Among recommendation types, IV to PO switch was the most accepted suggesting that clinicians are willing to perform switch even in hospitalized patients with cancer. Although IV to PO switch uptake was higher in the AMS PAF cohort, there was no reduction in LOS, hospital readmission or *C. difficile* infection as reported by other studies.^12^ Though not statistically significant, an association was observed between AMS PAF and 30-day unplanned hospital readmission. Our study did not collect the reason for hospital readmission; therefore, it is unknown what proportions were infection-related. This association may be in part due to baseline differences between the cohorts. The AMS PAF cohort had a higher proportion of patients with metastatic cancer who may be more likely to have unplanned admissions due to cancer complications. Further, the AMS PAF rounds select for those perceived to be more complex which can introduce unmeasured differences between cohorts.

Beyond early IV to PO switch it is also imperative to safely reduce overall antimicrobial exposure and related harms (eg, nausea/vomiting, *C. difficile* infection and antimicrobial resistance). Additionally, in patients with cancer, gastrointestinal microbiome dysbiosis is associated with adverse outcomes including decreased survival and higher rates of treatment-related adverse effects.^15^ At least two validated metrics for measuring antimicrobial consumption are recommended,^16^ we measured LOT, DOT and Inpatient DOT/Patient days. Despite duration recommendations being accepted in less than half of cases, we still observed lower antimicrobial consumption (LOT and inpatient DOT/patient days) in the AMS PAF cohort. This reduction was likely also driven by cessation and IV to PO switch recommendations. Although not statistically significant, there was an association between AMS PAF and 30-day antimicrobial recommencement, however despite this, overall antibiotic consumption remained lower. An immortal time bias may have contributed to the association, those who stopped antibiotics earlier had more opportunities to recommence. This bias likely disproportionately affected the AMS PAF cohort.

Our time-and-motion study findings are consistent with other published results.^17,18^ Applying the time-and-motion data to the retrospective review, we observed a mean 2-hour reduction in administration time per inpatient admission associated with AMS PAF. This time saving facilitates completion of other important clinical and administrative nursing duties thus improving efficiency of healthcare delivery. Additionally, decreased intravenous medication use reduces plastic waste promoting sustainable healthcare practices.^18^ This highlights an underrecognised benefit of AMS PAF.

We conducted a pragmatic study mostly utilizing existing data generated from routine AMS PAF processes; however, due to the nature of the study there are several limitations. The unblinded nature of the IV to PO switch assessments by the study team may introduce bias. Differences in baseline demographics and antimicrobial indications between cohorts were identified. For example, there was a higher proportion of antibiotics prescribed for urinary/gethird nitourinary infections in the AMS PAF cohort, whereas a higher proportion of intra-abdominal infections were observed in the treatment team-directed care cohort. In these scenarios, antimicrobial indication may have influenced clinician willingness to perform IV to PO switch. As discussed, a higher proportion of patients with metastatic cancer were observed in the AMS PAF cohort which may have affected the observed clinical outcomes. We adjusted for this using the Charlson Comorbidity Index; however, it is not validated as a measure of the likelihood of receiving prolonged intravenous antimicrobials.^19^ Additionally, since the resource-limited AMS team selectively prioritize complex patients for review, there may be other, unmeasured confounders. Despite these limitations, our study still supports IV to PO switch as a feasible and safe intervention in cancer cohorts.

## Conclusion

In a dedicated cancer cohort, AMS PAF was associated with earlier IV to PO switch and decreased antimicrobial consumption. There was no difference in clinical outcomes or LOS, suggesting safety of the approach despite reduced antimicrobial consumption. These findings support the feasibility and safety of implementing AMS PAF facilitated IV to PO switch protocols in oncology settings.

## Supporting information

10.1017/ash.2026.10880.sm001 Singh et al. supplementary material Singh et al. supplementary material

## Data Availability

Data are not publicly available due to patient privacy and ethics restrictions.

## Supplementary material

The supplementary material for this article can be found at https://doi.org/10.1017/ash.2026.10880

## Appendix A: Intravenous to oral switch criteria

**Table:**

**Inclusion**	**Exclusion**
Blood pressure and oxygenation stable or improving without additional support for ≥24 hours. Additional support defined as: • Fluid resuscitation • Vasopressors or inotropes • Respiratory ventilation	Awaiting surgical/procedural source control for known or suspected infection
Afebrile (36-38°C) for ≥24 hours	Awaiting investigations (eg CT scan) to rule out deep seated infection
Tolerating other oral fluids and medicines	Gastrointestinal absorption issues including: • Known or suspected ileus or obstruction • Known malabsorption syndrome • Proximal resection of small intestines • High (>500mL/day) nasogastric tube output (if applicable) • Active gastrointestinal bleed • History of bariatric surgery
Tolerating enteral feeding at goal rate (if applicable)	No oral regimen for directed therapy available

## Table 1. Long description

A table comparing patient characteristics between treatment team-directed care and AMS prospective audit and feedback cohorts. The table has 22 rows and 7 columns. The columns are labeled Treatment team-directed care, AMS PAF, and SMD. The rows are labeled with various patient characteristics and their corresponding values. Row 1: Hospital encounters (n), 112, 92. Row 2: Patients (n), 108, 87. Row 3: Antimicrobial prescriptions (n), 330, 224. Row 4: Age (years) (median [IQR]), 64.5 (53-72.5), 62.5 (47-72), 0.172. Row 5: Male n(%), 68 (60.7), 49 (53.3), 0.136. Row 6: Female n(%), 44 (39.3), 43 (46.7). Row 7: Metastatic cancer n(%), 63 (56.3), 63 (68.5), 0.234. Row 8: Charlson comorbidity index (median [IQR]), 7 (5-9), 7 (5-9), 0.013. Row 9: Medical oncology n(%), 56 (50.0), 59 (64.1), 0.288. Row 10: Lung, 17 (15.1), 10 (10.9), 0.136. Row 11: Sarcoma, 5 (4.5), 11 (12.0), 0.363. Row 12: Head and Neck, 8 (7.1), 7 (7.6), 0.028. Row 13: Other, 26 (23.2), 31 (33.7), 0.135. Row 14: Surgical n(%), 56 (50.0), 33 (35.9), 0.288. Row 15: Colorectal, 30 (26.8), 18 (19.6), 0.170. Row 16: Upper gastrointestinal, 9 (8.0), 4 (4.4), 0.145. Row 17: Skin, 4 (3.6), 1 (1.1), 0.174. Row 18: Sarcoma, 2 (1.8), 3 (3.3), 0.090. Row 19: Other, 11 (9.8), 7 (7.6), 0.078. Row 20: Antimicrobial at time of first review n(%), Meropenem, 1 (0.9), 4 (4.3), 0.214. Row 21: Antimicrobial at time of first review n(%), Piperacillin-tazobactam, 59 (52.7), 46 (50.0), 0.054. Row 22: Antimicrobial at time of first review n(%), Cefepime, 5 (4.5), 4 (4.3), 0.009. Row 23: Antimicrobial at time of first review n(%), Ceftriaxone, 28 (25.0), 23 (25.0), 0.000. Row 24: Antimicrobial at time of first review n(%), Amoxicillin-clavulanate (IV), 11 (9.8), 2 (2.2), 0.323. Row 25: Antimicrobial at time of first review n(%), Other, 8 (7.1), 13 (14.1), 0.220. Row 26: Antimicrobial indication at time of first review n(%), Respiratory tract, 104 (31.5), 72 (32.1), 0.013. Row 27: Antimicrobial indication at time of first review n(%), Intra-abdominal, 109 (33.0), 50 (22.3), 0.241. Row 28: Antimicrobial indication at time of first review n(%), Urinary/genitourinary, 16 (4.8), 28 (12.5), 0.273. Row 29: Antimicrobial indication at time of first review n(%), Fever or suspected infection (clinical syndrome not identified), 60 (18.2), 40 (17.9), 0.008. Row 30: Antimicrobial indication at time of first review n(%), Other, 41 (12.4), 34 (15.2), 0.081.

Navigate back to Table 1..

## Table 2. Long description

A table comparing antimicrobial consumption between treatment team-directed care and AMS PAF cohorts. The table has 10 rows and 5 columns. Column headers are Treatment team-directed care (n = 112 encounters), AMS PAF (n = 92 encounters), Adjusted β-coefficient (95% CI), and p-value. Row labels include LOT (IV + PO), PO, IV, DOT (IV + PO), PO, IV, Inpatient DOT/patient days (IV + PO), PO, and IV. Each row provides median values with interquartile ranges for both cohorts, followed by adjusted β-coefficient with 95% confidence intervals and p-values. Notable trends include significantly lower IV antimicrobial LOT and inpatient DOT/patient days in the AMS PAF cohort.

Navigate back to Table 2..

## Table 3. Long description

A table comparing clinical outcomes for treatment team-directed care and AMS PAF. The table has 6 rows and 5 columns. The columns are labeled Treatment team-directed care, AMS PAF, Unadjusted OR (95% CI), Adjusted oOR (95% CI), and p-value. The rows are labeled Mortality (30-day), ICU admission (30-day), Unplanned readmission (30-day), Antibiotic re-commencement (30-day), and C. difficile infection (90-day). Each row provides data for the respective clinical outcome, including the number of events and percentages for Treatment team-directed care and AMS PAF, unadjusted and adjusted odds ratios with 95% confidence intervals, and p-values.

Navigate back to Table 3..
