# Supplementary material for: Antimicrobial stewardship prospective audit and feedback improves uptake of intravenous to oral antimicrobial switch in oncology inpatients: a cohort study
Source: Antimicrob Steward Healthc Epidemiol. 2026 Oct 5;6(1):e310. doi: 10.1017/ash.2026.10880 (PMC13760401; doi:10.1017/ash.2026.10880)
Supplement: Supplementary file 1 [file S2732494X26108808sup001.docx]

**Title:** Antimicrobial stewardship prospective audit and feedback improves uptake of intravenous to oral antimicrobial switch in oncology inpatients: a cohort study

**Authors:** Nikhil R Singh, A/Prof. Tim Spelman, Jacob Howell, Chulong Luo, Kanon Machray, Rosie Tran, A/Prof Safeera Y Hussainy, Chiao X Lim, Senthil Lingaratnam, Abby P Douglas, Prof. Karin A Thursky, A/Prof. Gabrielle M Haeusler

Table 1. Antimicrobial consumption (stabilised inverse probability of treatment weight sensitivity analysis)

| **Antimicrobial Consumption** | **Weighted control mean** | **Weighted AMS PAF mean** | **Mean difference** | **95% CI lower** | **95% CI upper** | **p-value** |
| --- | --- | --- | --- | --- | --- | --- |
| LOT (IV + PO) | 15.603 | 11.807 | -3.796 | -7.123 | -0.470 | **0.025** |
| LOT (PO) | 8.179 | 6.373 | -1.806 | -4.240 | 0.628 | 0.146 |
| LOT (IV) | 8.411 | 6.025 | -2.386 | -4.081 | -0.691 | **0.006** |
| DOT (IV + PO) | 14.778 | 10.779 | -3.999 | -7.225 | -0.774 | **0.015** |
| DOT (PO) | 4.237 | 3.293 | -0.944 | -2.561 | 0.672 | 0.252 |
| DOT (IV) | 10.522 | 7.486 | -3.036 | -5.570 | -0.502 | **0.019** |
| Inpatient DOT/patient days (IV + PO) | 1.159 | 0.987 | -0.172 | -0.338 | -0.005 | **0.044** |
| Inpatient DOT/patient days (IV) | 0.863 | 0.674 | -0.190 | -0.321 | -0.058 | **0.005** |
| Inpatient DOT/patient days (PO | 0.294 | 0.314 | 0.020 | -0.086 | 0.126 | 0.714 |

*Abbreviations: LOT – length of therapy; DOT – days of therapy; AMS PAF – antimicrobial stewardship prospective audit and feedback*

Table 2. IV to PO switch and clinical outcomes (stabilised inverse probability of treatment weight sensitivity analysis)

| **Binary outcome** | **Weighted control risk** | **Weighted AMS PAF risk** | **Risk ratio** | **95% CI lower** | **95% CI upper** | **p-value** |
| --- | --- | --- | --- | --- | --- | --- |
| IV to PO | 0.173 | 0.264 | 1.527 | 0.897 | 2.602 | 0.119 |
| 30-day mortality | 0.167 | 0.174 | 1.042 | 0.566 | 1.918 | 0.896 |
| 30-day ICU admission | 0.040 | 0.035 | 0.882 | 0.216 | 3.608 | 0.861 |
| 30-day unplanned readmission | 0.123 | 0.229 | 1.866 | 0.977 | 3.564 | 0.059 |
| Antibiotic recommencement within 30 days | 0.188 | 0.305 | 1.623 | 0.967 | 2.725 | 0.067 |

*Abbreviations*: ICU – intensive care unit, AMS PAF – antimicrobial stewardship prospective audit and feedback
